# Complete chloroplast genome of *Adonis pseudoamurensis* W.T.Wang (Ranunculaceae)

**DOI:** 10.1080/23802359.2023.2256493

**Published:** 2023-09-15

**Authors:** Xiao-Yan Zhang, Zhao-Lei Zhang, Li-Qiu Zhang, Li-Fan Zhang, Jun-Yi Zhu, Chang-Song Xue

**Affiliations:** a School of Food Science and Engineering, Tonghua Normal University, Tonghua, P.R. China; b School of Medicine and Pharmacy, Tonghua Normal University, Tonghua, P.R. China; c School of Life Science, Tonghua Normal University, Tonghua, P.R. China; d Hebei Key Laboratory of Study and Exploitation of Chinese Medicine, Chengde Medical University, Chengde, P.R. China

**Keywords:** *Adonis pseudoamurensis*, complete chloroplast genome, phylogenetic analysis, ranunculaceae

## Abstract

*Adonis pseudoamurensis* W.T. Wang 1980 is an important traditional medicinal plant used for the treatment of cardiac diseases. The complete chloroplast (cp) genome of *Adonis pseudoamurensis* is reported for the first time in this study. The circular cp genome is 156,917 bp in length, consisting of a large single-copy region (86,262 bp), a small single-copy region (18,067 bp), and two inverted repeat regions (26,294 bp). The genome encodes 129 genes, comprising 84 protein-coding genes, 37 transfer RNA (tRNA) genes, and 8 ribosomal RNA (rRNA) genes. Phylogenetic analysis showed that *A. pseudoamurensis* is closely related to *A. amurensis*.

## 1. Introduction

The genus *Adonis* (Ranunculaceae) comprises 32 annual or perennial herbaceous species (Son, 2015). Owing to their complex chemical composition and cardiac-enhancing effects, species in *Adonis* are commonly used in folk medicine in Europe and China (Hao et al. 2015; Kaneko et al. 2008; Shang et al. 2019). *Adonis pseudoamurensis* W.T. Wang 1980 is an early spring flowering species mainly distributed in northeast China (Yang et al. 2015). This species is morphologically similar to *A. amurensis* but differs from it in having sessile cauline leaves and ovate to rhomboid sepals (Wang and Liu, 1988; Wang 1994a, 1994b). Based on internal transcriptional spacer sequence (ITS) and chloroplast sequences independent phylogenetic analyses of the genus *Adonis* confirmed that present *Adonis* taxa were genotypically differentiated according to their length of life and general morphology (Karahan et al. 2022; Seidl et al. 2022). *A. pseudoamurensis* has been synonymized with *A. ramosa* in Japan. However, similar morphological characteristics shared by the members of the genus *Adonis* render morphology-based identification of the species in the genus difficult (Suh et al. 2002). Therefore, it is necessary to distinguish *A. pseudoamurensis* from its closely related species in the genus *Adonis* with the help of molecular data. In this study, we sequenced the entire chloroplast genome of *A. pseudoamurensis* to provide useful information for the taxonomic and phylogenetic study of genus *Adonis*.

## 2. Materials and methods

**Figure 1.**
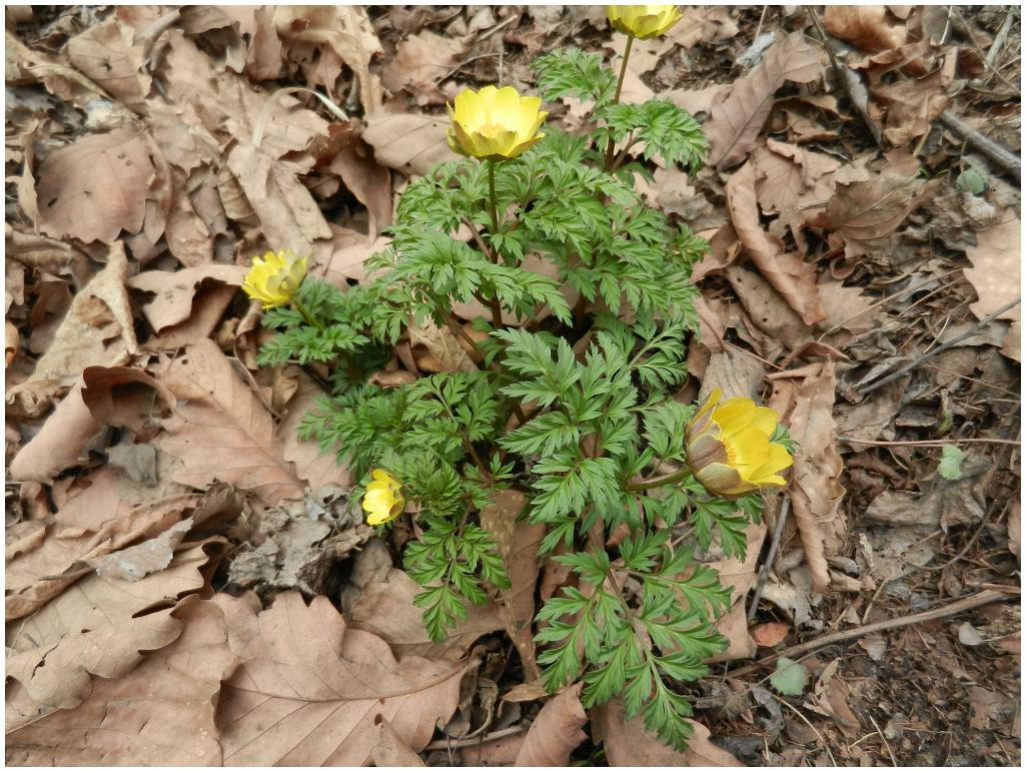
*Adonis pseudoamurensis* W.T. Wang 1980. This photo was taken by the author of this article, Li-Qiu Zhang. Herbs perennial, with two- to three-lobed leaves and flowers borne singly at the tips of stems or branches. Petals ca. 13, yellow; sepals purplish, about 1/2 as long as petals.

Fresh leaves of wild *A. pseudoamurensis* were collected from the campus of Tonghua Normal University (41°44′33.10″N, 125°56′44.28″E) and stored in silica gel (Figure 1). A voucher specimen was deposited in the Herbarium of Tonghua Normal University under the number sfxyyhsljcjzh20200429 (Liqiu Zhang, 13717943@qq.com). Total genomic DNA was extracted from silica-dried leaves using a modified CTAB method (Doyle and Doyle, 1987). DNA library preparation and paired-end read sequencing were performed on the Illumina NovaSeq 6000 platform. The cp genome was assembled using NOVOPlasty (NOVOPlasty4.2.pl -k) (Dierckxsens et al. 2017), and the *A. sutchuenensis* cp genome was used as the reference (Zhang et al. 2021). The depth of coverage was calculated by mapping the reads onto the chloroplast genome sequence with bowtie2 v2.3.4.3 (bowtie2 -x −1 -2 -S –sensitive-local -p 32) to determine the correctness of the assembly (Langmead and Salzberg, 2012). Chloroplast genome annotation was performed using the online web server CPGVAS2 (https://irscope.shinyapps.io/Chloroplot/) (Shi et al. 2019). The coding sites and codons of the predicted genes were manually corrected, and Geneious R11 (Biomatters Ltd., Auckland, New Zealand) was used to inspect the cp genome structure. The complete cp genome sequence was deposited in GenBank (accession number: MZ197990).

## 3. Result

**Figure 2.**
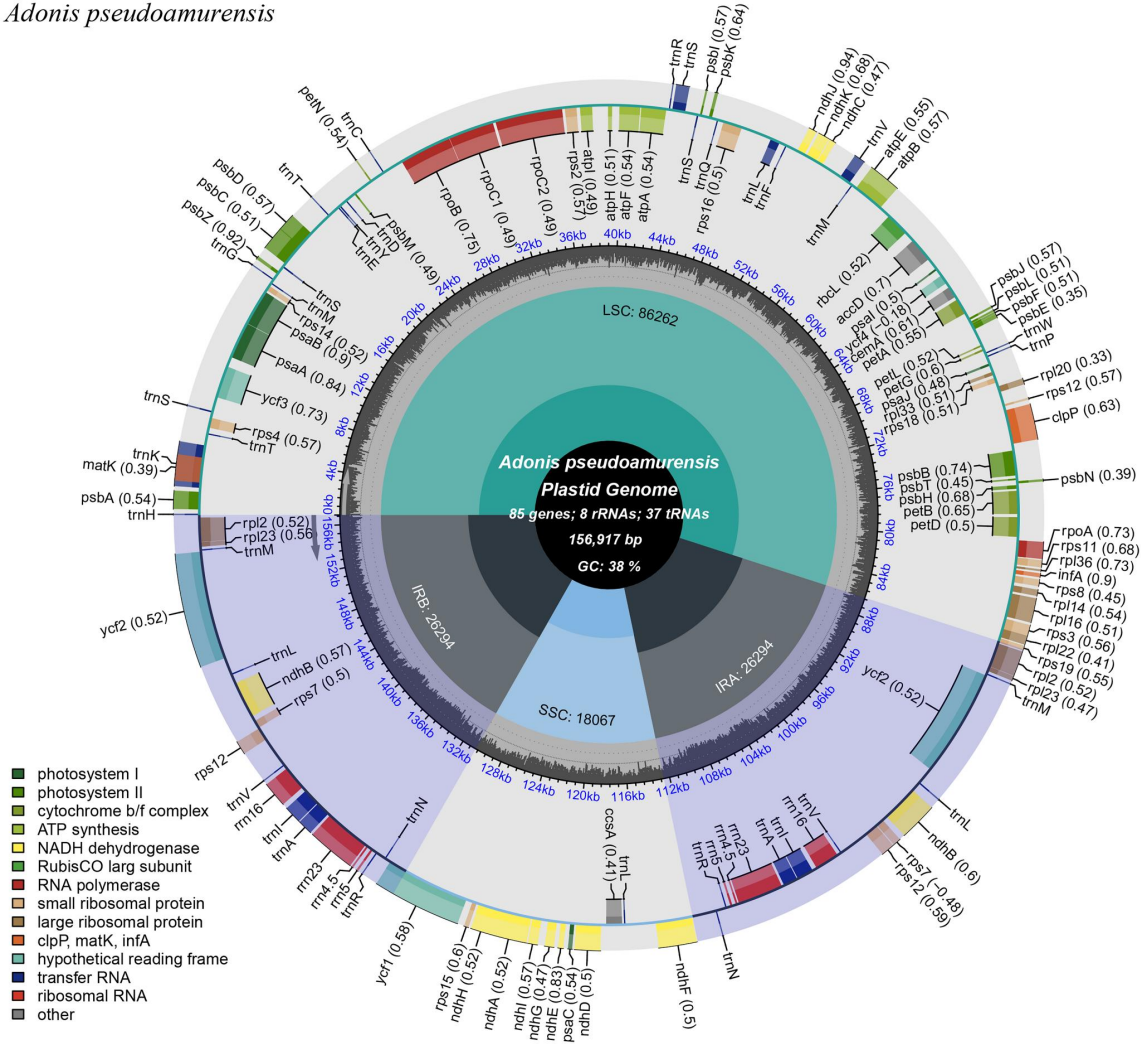
Chloroplast genome map of *A. pseudoamurensis*. Genes drawn outside the outer circle are transcribed counterclockwise, and genes drawn inside the outer circle are transcribed clockwise. Genes belonging to different functional groups are color-coded. The different colored legends in the bottom left corner indicate genes with different functions. The dark grey inner circle indicates the GC content of the chloroplast genome and the presence of nodes in the LSC, SSC, IR regions.

The complete cp genome of *A. pseudoamurensis* was 156,917 bp in length with an average depth of 337.62 X, and it included an 86,262 bp large single-copy region (LSC); an 18,067 bp small single-copy region (SSC); and two 26,294 bp inverted repeats (IRs) (Supplementary Figure 1). The GC content (%) of the whole genome, LSC, SSC, and IR regions was 37.95%, 36.14%, 31.55%, and 43.11%, respectively. A total of 129 genes were annotated, comprising 84 protein-coding genes, 8 rRNA genes, and 37 tRNA genes. Furthermore, 17 genes—seven tRNA, four rRNA, and six protein-coding genes—were repeated in the IR regions (Figure 2). Gene structure analysis was done for *ycf*3*, rpo*C1*, atp*F*, rps*16*, clp*P*, pet*B*, pet*D*, rpl*16*, rpl*2*, ndh*B*, ndh*A difficult to annotate genes (Supplementary Figure 2). The LSC region contained 61 protein-coding and 22 tRNA genes, whereas the SSC region contained one tRNA gene and 11 protein-coding genes. Nineteen genes (11 protein-coding and eight tRNA genes) contained one intron, and two genes (*ycf*3 and *clp*P) had two introns. The *rps*12 gene is a trans-spliced gene, with the 5' end located in the LSC region and the copied 3' end located in the IR region.

**Figure 3.**
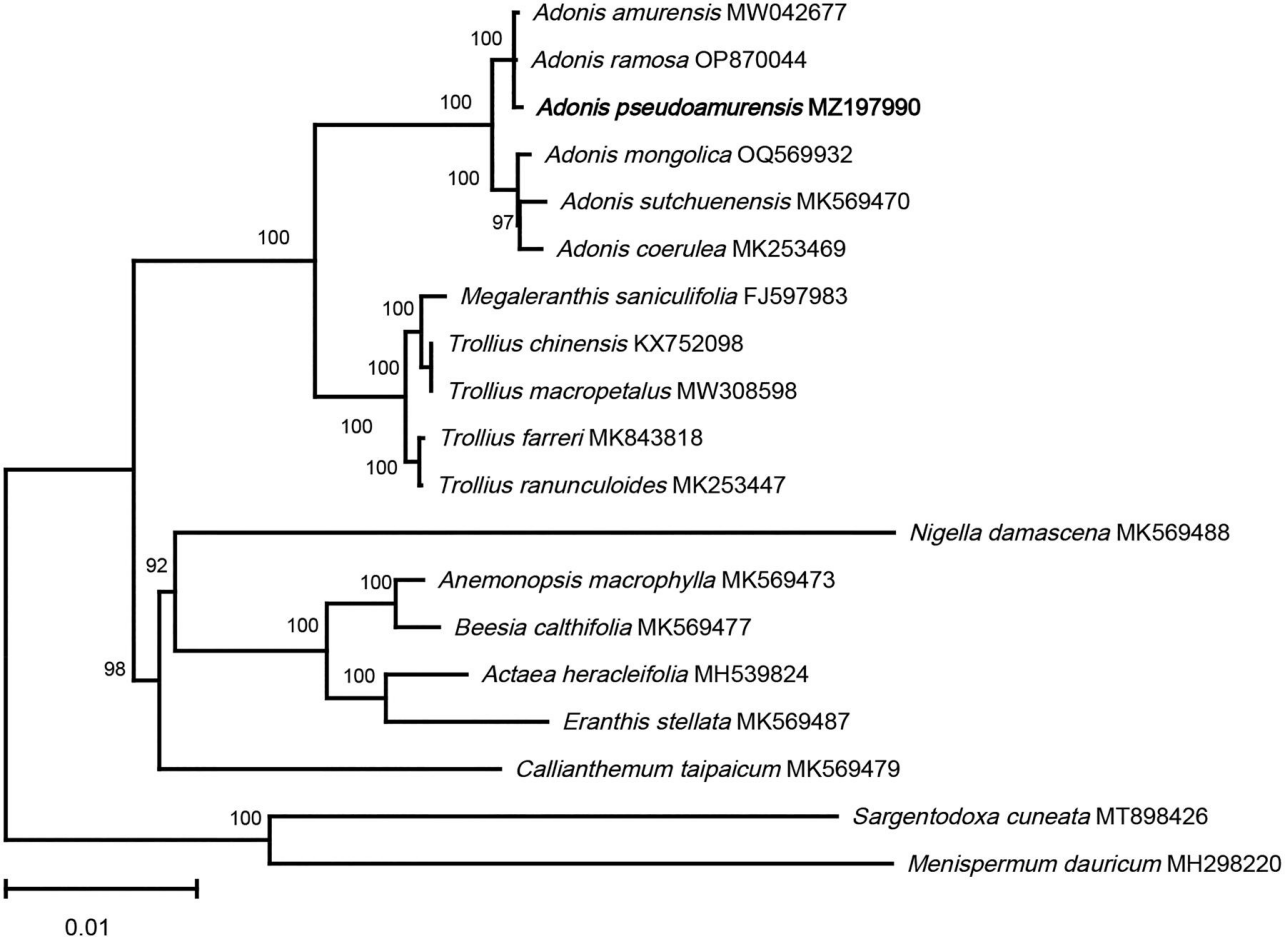
The ML phylogenetic tree is based on chloroplast genome sequences of *Adonis pseudoamurensis* species from the Ranunculaceae family, with *Sargentodoxa cuneata* (MT898426) and *Menispermum dauricum* (MH298220) as outgroups. Support values above the branches are ML bootstrap support. The following sequences were used: *Trollius ranunculoides* MK253447, *Adonis coerulea* MK253469 (He et al. 2019), *Adonis pseudoamurensis* MZ197990, *Adonis ramosa OP870044*, *Megaleranthis saniculifolia* FJ597983 (Kim et al. 2009), *Trollius chinensis* KX752098, *Adonis sutchuenensis* MK569470, *Callianthemum taipaicum* MK569479, *Anemonopsis macrophylla* MK569473, *Beesia calthifolia* MK569477, *Eranthis stellata* MK569487, *Nigella damascena* MK569488 (Zhai et al. 2019), *Actaea heracleifolia* MH539824 (Park et al. 2018), *Menispermum dauricum* MH298220 (Hina et al. 2018), *Trollius farreri* MK843818 (Yu et al. 2019), *Adonis amurensis* MW042677, *Sargentodoxa cuneata* MT898426 (Cui et al. 2021), *Trollius macropetalus* MW308598.

To elucidate the phylogenetic relationship between *A. pseudoamurensis* and other species of Ranunculaceae, 16 complete chloroplast genome sequences of Ranunculaceae were downloaded from NCBI, and the phylogenetic tree (Figure 3) was constructed using the maximum likelihood (ML) method implemented in FastTree 2.1.11 software (Price et al. 2009) with 1000 bootstrap replicates. *Sargentodoxa cuneata* (MT898426), and *Menispermum dauricum* (MH298220) were selected as outgroup species. In the ML tree, the four *Adonis* species are perennial species and clustered together in a well-supported clade (bootstrap support = 100%). *A. pseudoamurensis* and *A. amurensis* formed a highly supported clade (bootstrap support = 100%) that was sister to *A. sutchuenensis*, confirming the close phylogenetic relationship between *A. pseudoamurensis* and *A. amurensis*. The chloroplast genome sequence of *A. pseudoamurensis* provided here is a useful resource for further phylogenetic and taxonomic studies and species identification in the genus *Adonis*.

## 4. Discussion and conclusion

In this study, the chloroplast genome sequence of *A. pseudoamurensis* was assembled for the first time and the structure of this species was annotated. This phylogenetic result is similar to the phylogenetic analysis of the ribosomal ITS in *A. pseudoamurensis* (Suh et al. 2002). Comparison of the *A. pseudoamurensis* plastome to previously published data shows a high level of gene synteny with publicly available *A. amurensis* and *A ramosa*. This study provided useful information on the taxonomic and phylogenetic study of the genus *Adonis*.

## Ethical approval

The material involved in the article does not involve ethical conflicts. This species is neither endangered on the CITES catalogue nor collected from a natural reserve, so it did not need specific permissions or licenses. All collection and sequencing work was strictly executed under local legislation and related laboratory regulations to protect wild resources.

## Supplementary Material

Supplemental Material

Supplemental Material

Supplemental Material

Supplemental Material

## Data Availability

The data that support the findings of this study are openly available in GenBank of NCBI at https://www.ncbi.nlm.nih.gov, reference number MZ197990. The associated BioProject, SRA, and Bio-Sample numbers are PRJNA878453, SRR21533504, and SAMN30726550, respectively.
