# Supplementary figures and images for: Complete chloroplast genome of Adonis pseudoamurensis W.T.Wang (Ranunculaceae)
Source: Mitochondrial DNA B Resour. 2023 Sep 15;8(9):981–4. doi: 10.1080/23802359.2023.2256493 (PMC10506428; doi:10.1080/23802359.2023.2256493)

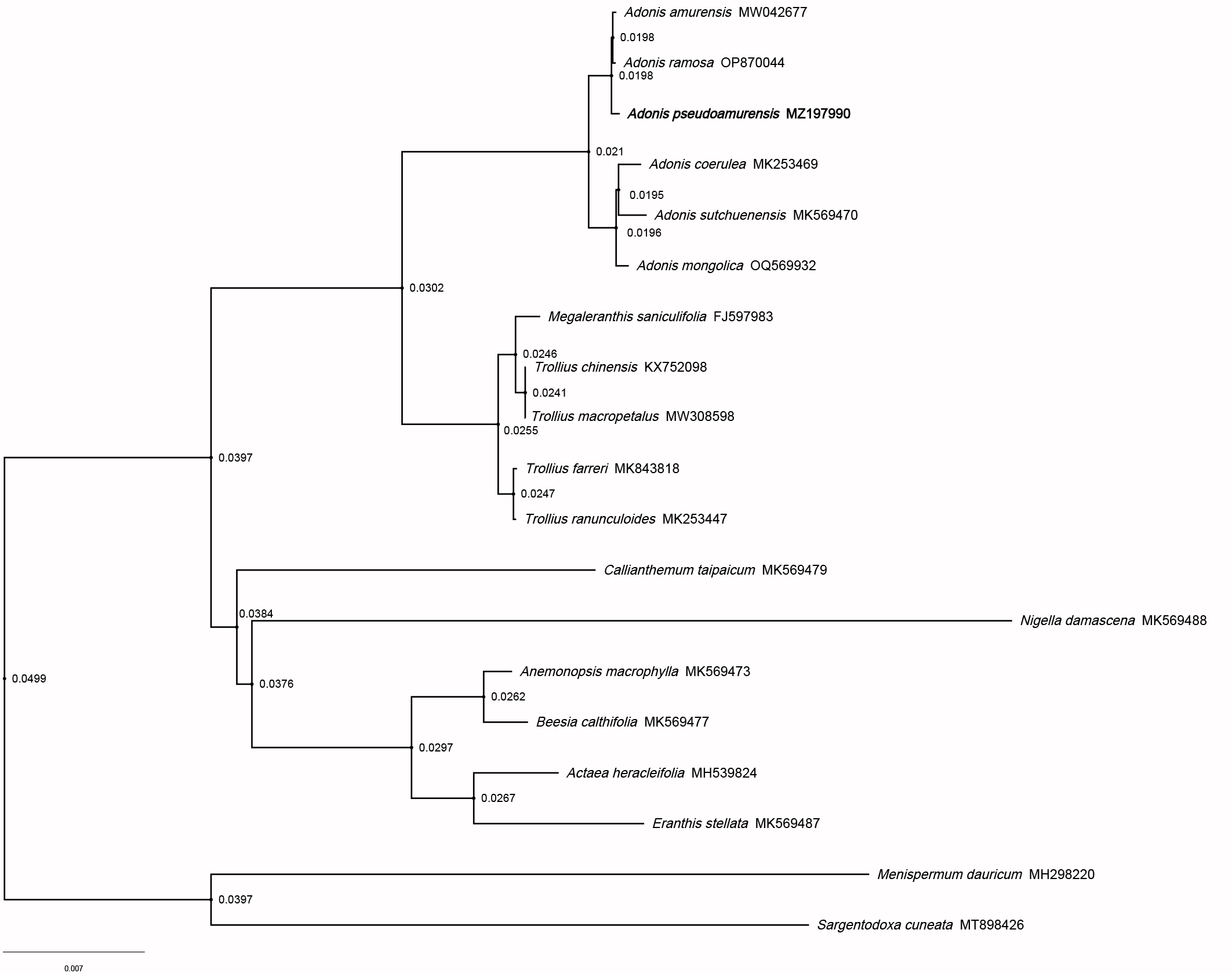

Supplement: Supplemental Material [file TMDN_A_2256493_SM7248.tif]

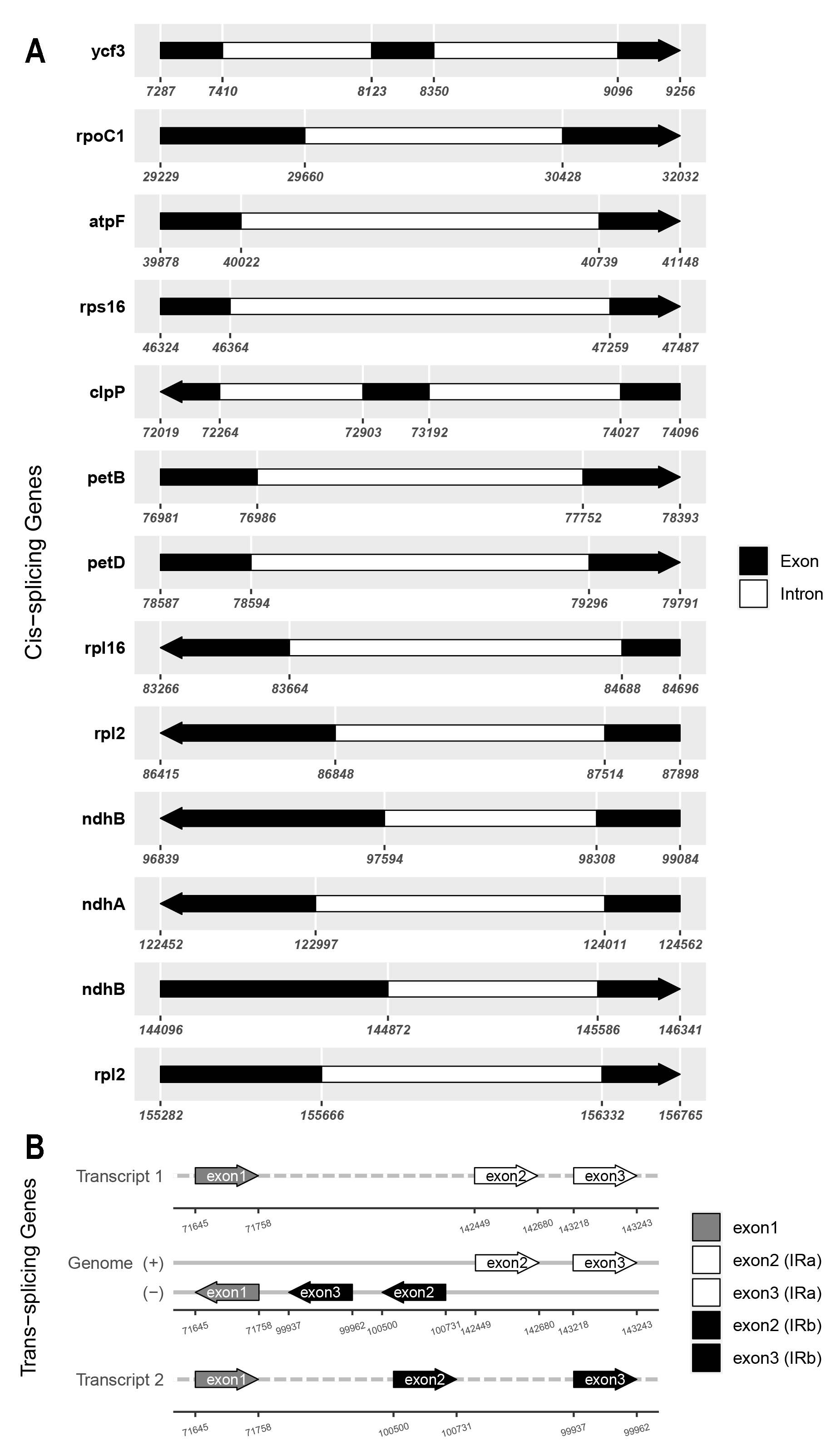

Supplement: Supplemental Material [file TMDN_A_2256493_SM7247.tif]

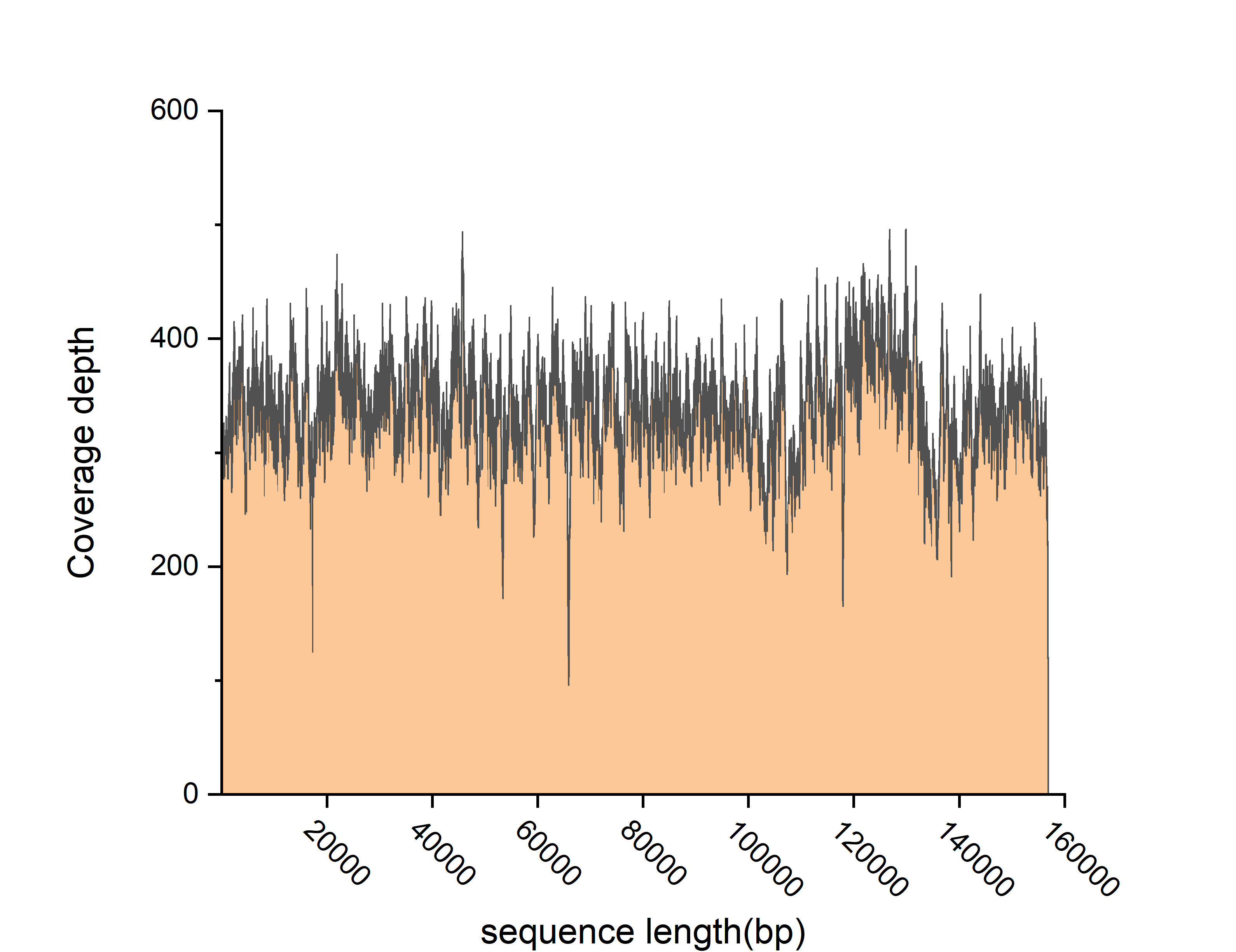

Supplement: Supplemental Material [file TMDN_A_2256493_SM7243.tif]
