# Supplementary material for: Complete chloroplast genome of Adonis pseudoamurensis W.T.Wang (Ranunculaceae)
Source: Mitochondrial DNA B Resour. 2023 Sep 15;8(9):981–4. doi: 10.1080/23802359.2023.2256493 (PMC10506428; doi:10.1080/23802359.2023.2256493)
Supplement: Supplemental Material [file TMDN_A_2256493_SM7239.docx]

**Complete chloroplast genome of *Adonis pseudoamurensis* W.T.Wang ([Ranunculaceae](https://www.ncbi.nlm.nih.gov/pubmed/30826488))**

Xiao-Yan Zhang^1^, Zhao-Lei Zhang^4^, Li-Qiu Zhang^2^, Li-Fan Zhang^3^, Jun-Yi Zhu^3^, Chang-Song Xue^2^*^*^*

*^1^School of food science and engineering, Tonghua Normal University, Tonghua 134002, China*

*^2^School of Medicine and Pharmacy, Tonghua Normal University, Tonghua 134002, China*

*^3^School of Life Science, Tonghua Normal University, Tonghua 134002, China*

*^4^Hebei Key Laboratory of Study and Exploitation of Chinese Medicine, Chengde Medical University, Chengde, 067000, China*

***^*^*Correspondence:** Chang-Song Xue, E-mail: [799155778@qq.com](mailto:799155778@qq.com)


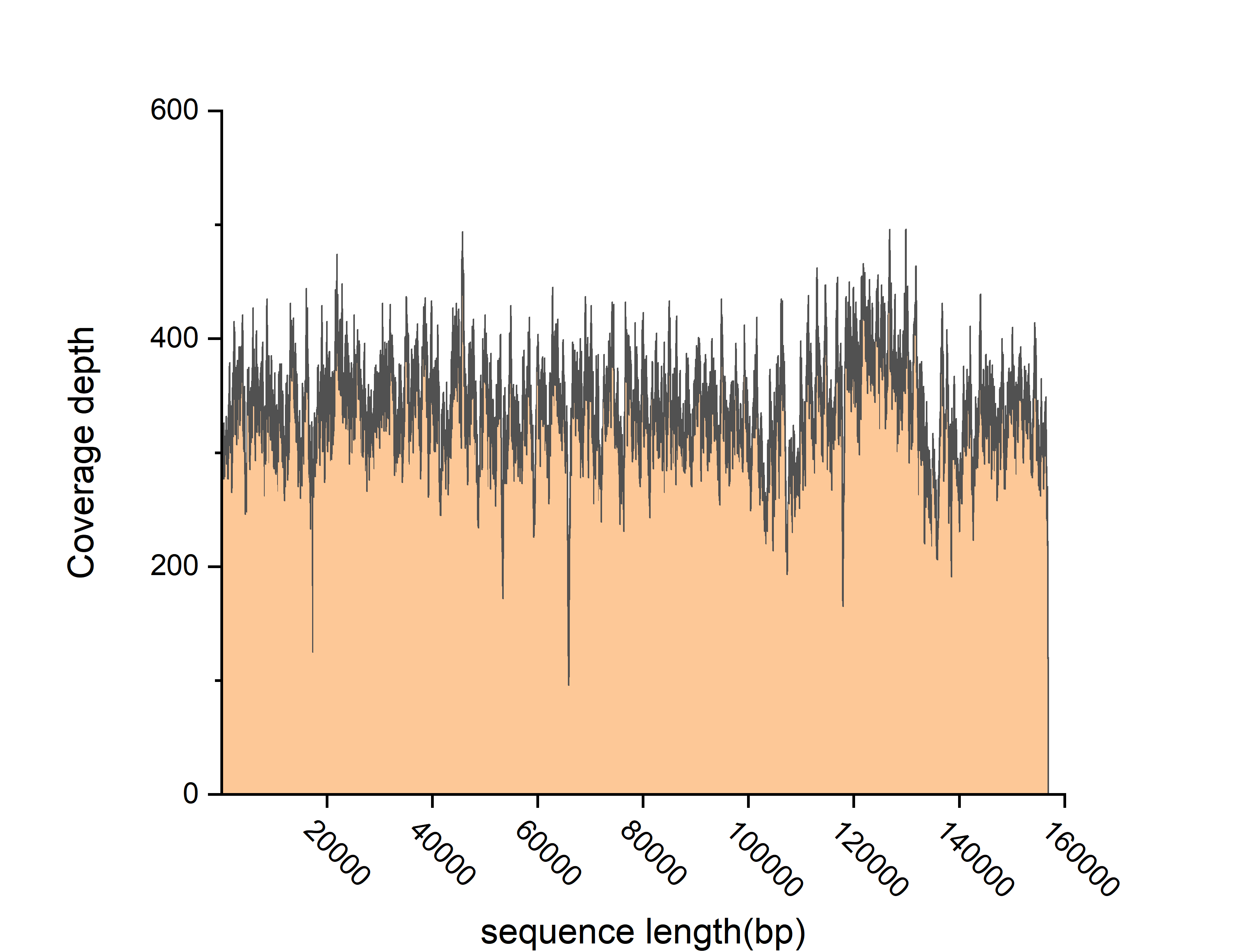


**Supplementary Figure 1** Coverage depth figure of the *Adonis pseudoamurensis* chloroplast genome. The horizontal coordinate is the base of the chloroplast genome and the vertical coordinate is the depth of sequencing corresponding to that base.


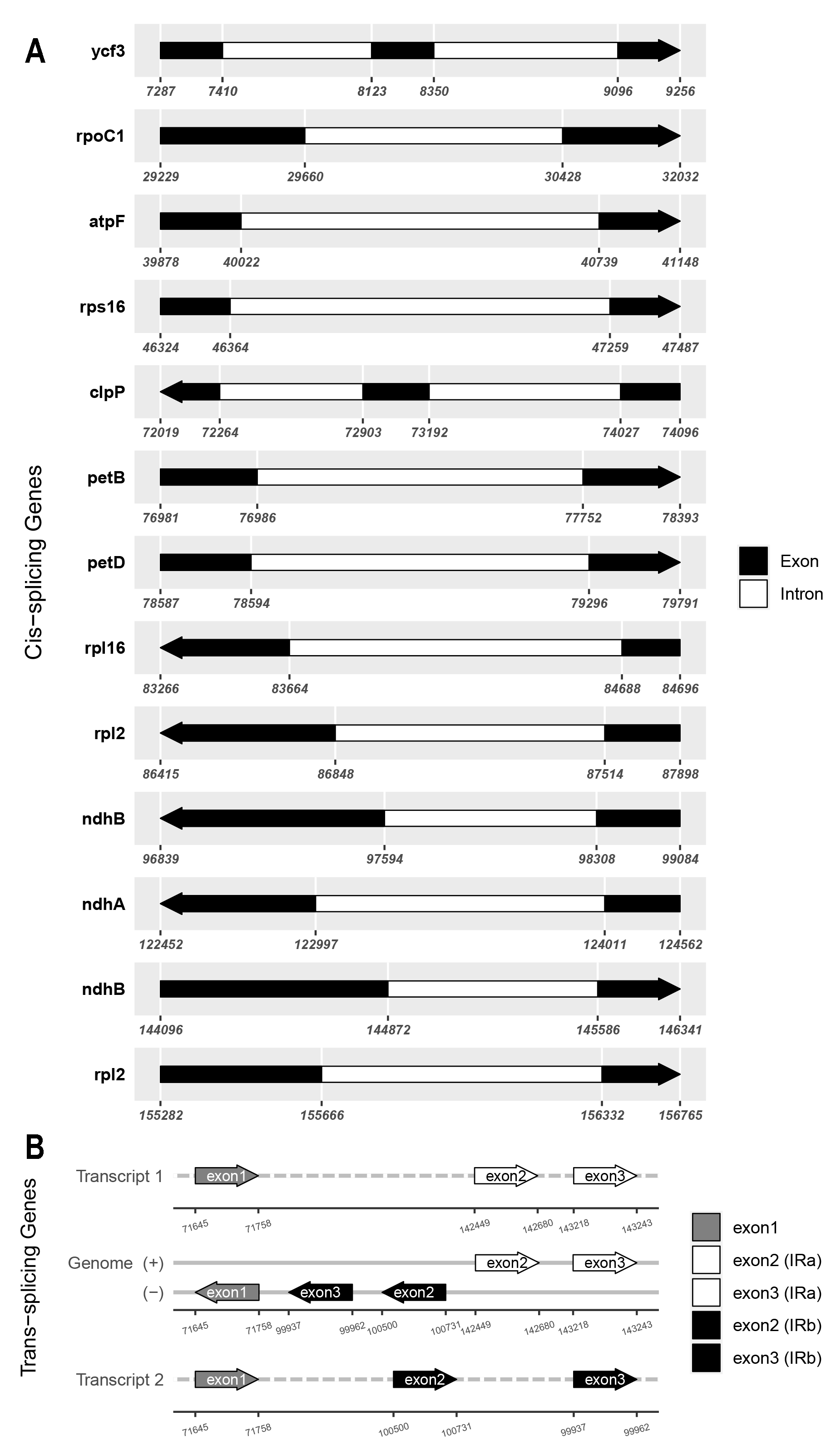


**Supplementary Figure 2** A. Schematic map of the cis-splicing genes in the *A. pseudoamurensis* chloroplast genome. B. Schematic map of the trans-splicing gene rps12 in the chloroplast genome.


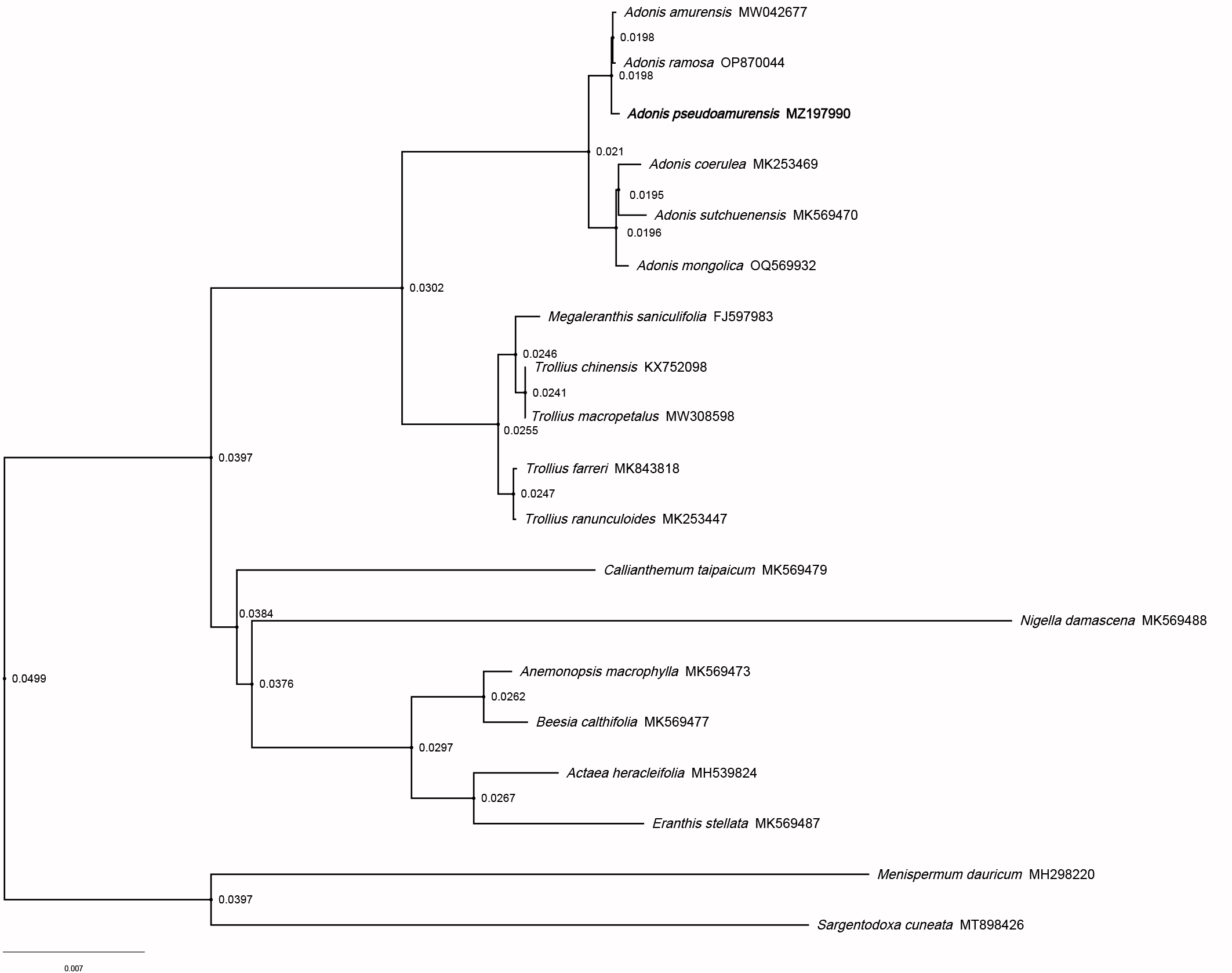


**Supplementary Figure 3** The BI phylogenetic tree based on chloroplast genome sequences of Adonis pseudoamurensis species from the Ranunculaceae family, with Sargentodoxa cuneata MT898426 and Menispermum dauricum MH298220 as outgroups. Support values above the branches are BI bootstrap support.
